# Supplementary material for: Increasing rice yield with low ammonia volatilization by combined application of controlled-release blended fertilizer and densification
Source: PLoS One. 2025 Feb 10;20(2):e0318177. doi: 10.1371/journal.pone.0318177 (PMC11809907; doi:10.1371/journal.pone.0318177)
Supplement: S1 Table — (DOCX) [file pone.0318177.s001.docx]

**Supporting Information**

**Table S1: The cost of agricultural materials, labor, and seedlings**

| Year | Rice purchase price（Yuan · kg^-1^） | Urea（Yuan  · t^-1^） | Coated urea（Yuan · t^-1^） | Calcium bicarbonate（Yuan · t^-1^） | Potassium sulfate（Yuan · t^-1^） | Insecticide（Yuan · ha^-1^） | Herbicide（Yuan · ha^-1^） |
| --- | --- | --- | --- | --- | --- | --- | --- |
| 2021 | 2.80 | 2500 | 3300 | 1200 | 3200 | 300 | 400 |
| 2022 | 2.81 | 2500 | 3300 | 1200 | 3200 | 300 | 400 |
| Year | Plough（Yuan · ha^-1^） | seedling transplanting（Yuan · ha^-1^） | spread manure（Yuan · ha^-1^） | spray insecticide（Yuan · ha^-1^） | Harvest（Yuan · ha^-1^） | cost of conventional（Yuan · ha^-1^） | densified seedlings（Yuan · ha^-1^） |
| 2021 | 1200 | 600 | 360 | 360 | 800 | 1350 | 1755 |
| 2022 | 1200 | 600 | 360 | 360 | 800 | 1350 | 1755 |
